# Increased iNKT17 Cell Frequency in the Intestine of Non-Obese Diabetic Mice Correlates With High *Bacterioidales* and Low *Clostridiales* Abundance

**DOI:** 10.3389/fimmu.2018.01752

**Published:** 2018-07-30

**Authors:** Lorena De Giorgi, Chiara Sorini, Ilaria Cosorich, Roberto Ferrarese, Filippo Canducci, Marika Falcone

**Affiliations:** ^1^Experimental Diabetes Unit, Division of Immunology, Transplantation and Infectious Diseases, IRCCS San Raffaele Scientific Institute, Milan, Italy; ^2^Department of Medicine, Karolinska Institute, Stockholm, Sweden; ^3^Department of Biotechnology and Life Sciences, University of Insubria, Varese, Italy

**Keywords:** natural killer T cells, interleukin-17, dendritic cells, microbiota, autoimmune diabetes

## Abstract

iNKT cells play different immune function depending on their cytokine-secretion phenotype. iNKT17 cells predominantly secrete IL-17 and have an effector and pathogenic role in the pathogenesis of autoimmune diseases such as type 1 diabetes (T1D). In line with this notion, non-obese diabetic (NOD) mice that spontaneously develop T1D have an increased percentage of iNKT17 cells compared to non-autoimmune strains of mice. The factors that regulate iNKT cell expansion and acquisition of a specific iNKT17 cell phenotype are unclear. Here, we demonstrate that the percentage of iNKT17 cells is increased in the gut more than peripheral lymphoid organs of NOD mice, thus suggesting that the intestinal environment promotes iNKT17 cell differentiation in these mice. Increased intestinal iNKT17 cell differentiation in NOD mice is associated with the presence of pro-inflammatory IL-6-secreting dendritic cells that could contribute to iNKT cell expansion and iNKT17 cell differentiation. In addition, we found that increased iNKT17 cell differentiation in the large intestine of NOD mice is associated with a specific gut microbiota profile. We demonstrated a positive correlation between percentage of intestinal iNKT17 cells and bacterial strain richness (α-diversity) and relative abundance of *Bacterioidales* strains. On the contrary, the relative abundance of the anti-inflammatory *Clostridiales* strains negatively correlates with the intestinal iNKT17 cell frequency. Considering that iNKT17 cells play a key pathogenic role in T1D, our data support the notion that modulation of iNKT17 cell differentiation through gut microbiota changes could have a beneficial effect in T1D.

## Introduction

Vα14iNKT cells play pleiotropic functions in the immune system that either boost or dampen T and B cell immunity in infections, antitumor responses, and autoimmune diseases (1, 2). These opposite immunological functions are mediated by iNKT cells with different cytokine-secretion phenotype. In fact, iNKT cells are classified into iNKT1, iNKT2, and iNKT17 based on their release of Th1, Th2, and Th17 cytokines. iNKT1 cells that predominantly secrete IFN-γ play an effector adjuvant function that enhances T cell responses and is fundamental to clear infections and tumors while iNKT2 cells releasing IL-4 and IL-13 mainly provide B cell help and are involved in allergic reactions. The iNKT17 cells have been recently characterized and they also play effector functions in infections (3), asthma (4), and autoimmune diseases like collagen-induced arthritis (5) and autoimmune type 1 diabetes (T1D) (6, 7). In fact, non-obese diabetic (NOD) mice that spontaneously develop T1D have an increased frequency and absolute number of iNKT17 cells (1) that are directly responsible for triggering autoimmune diabetes (6).

The factors that regulate iNKT cell expansion and acquisition of a specific cytokine-secretion phenotype and function are still largely unknown. Most iNKT cells are already committed toward a specific cytokine-secretion phenotype when they exit the thymus (8). However, it is now clear that the iNKT cell repertoire can be expanded and modulated in the periphery and recent evidence indicates that the intestinal environment and the microbiota composition are instrumental to control iNKT cell expansion and acquisition of a specific cytokine-secretion phenotype (9). In support to this notion, lack of commensal gut microbiota in germ-free mice leads to less mature and hyporesponsive iNKT cells (10, 11). Moreover, housing conditions and the resulting differences in the gut microbiota composition alter iNKT cell functional maturation, release of cytokines, and acquisition of effector functions (10). The relative abundance of some gut commensal microbes plays a direct effect on iNKT cells. For example, *Bacteroides fragilis* limits iNKT cell expansion in the gut mucosa by providing inhibitory sphingolipid antigens that bind the iNKTCR (12). On the other hand, some lipids derived from *Sphingomonas* species are capable to activate iNKT cells and promote their intestinal expansion (13, 14) and functional maturation (10). Commensal microbiota can also influence iNKT cells through antigen-independent mechanism such as epigenetic regulation of CXCL16 expression that promotes iNKT cell recruitment to the gut mucosa (11). Although these findings demonstrate that commensal microbiota influence iNKT cell number and function, the capacity of the gut environment to drive iNKT cells toward a specific cytokine-secretion phenotype is yet to be determined.

Here, we show that NOD mice have increased iNKT17 cell frequency in the intestinal mucosa that is more evident than in peripheral lymphoid organs and liver. Moreover, we found that the augmented iNKT17 cell percentages correlate with a specific gut microbiota profile characterized by high bacterial richness, increased relative abundance of *Bacteroidales*, and reduction of *Clostridiales* strains.

## Materials and Methods

### Mice

Females NOD mice were purchased from Charles River Laboratories (Calco, Italy). In some experiments mice received antibiotic treatment (ampicillin, 1 g/L; neomycin 1 g/L; metronidazole, 1 g/L; vancomycin, 0.5 g/L) for 1 week in drinking water. All mice were maintained under specific pathogen-free conditions in the animal facility at San Raffaele Scientific Institute, and all experiments were conducted in accordance with the Institutional Animal Care and Use Committee (IACUC) according with the rules of the Italian Ministry of Health.

### Cell Isolation

Mononuclear cells were isolated from intestinal tissues as previously described (15, 16). After removal of the Peyer’s patches, small and large intestines were flushed with PBS, opened longitudinally, and predigested with 5 mM EDTA and 1 mM DTT for 20 min at 37°C. After removing epithelial cells and adipose tissue, the intestine was cut into small pieces and incubated in HBSS containing 0.5 mg/mL collagenase D, 1 mg/mL dispase II (Roche Diagnostics GmbH, Mannheim, Germany), and 5 U/mL DNase I (Sigma-Aldrich, St. Louis, MO, USA) for 20 min at 37°C. Digested tissues were washed, suspended in 5 mL of 40% Percoll (Sigma-Aldrich, St. Louis, MO, USA), and overlaid on 2.5 mL of 80% Percoll solution. Percoll gradient separation was performed by centrifugation at 1,000 *g* for 20 min at 20°C, and cells at the interface were collected. For lymphocyte isolation from liver, the total organ was meshed and hepatocytes were removed by Percoll gradient centrifugation. Splenocytes and lymph node cells were isolated by mechanical disruption of the tissues.

### Flow Cytometry

Single cell suspensions were stained for 20 min at 4°C with the following fluorochrome-conjugated monoclonal antibodies or tetramers in FACS buffer (PBS with 5% FBS, 0.1% sodium azide): PE anti-mouse αGalCer-loaded CD1d tetramers (PBS57/Dimerix from the NIH Tetramer Facility, Washington, DC, USA), FITC anti-mouse TCRβ, PerCP anti-mouse CD4, Pacific Blue CD8, APC-Cy7 anti-mouse CD3 (BD Biosciences, San Diego, CA, USA). For intracellular cytokine staining, single-cell suspensions were stimulated for 2.5 h with 50 ng/mL phorbol 12-myristate 13-acetate (PMA) and 1 µg/mL ionomycin (both from Sigma-Aldrich, St. Louis, MO, USA) in the presence of 10 µg/mL Brefeldin A (Sandoz, Princeton, NJ, USA). Cells were collected and labeled for iNKT cell surface markers and then fixed and permeabilized with fixation and permeabilization buffer (BD Biosciences, San Diego, CA, USA) and stained with PE-Cy7 anti-mouse IL-17 and FITC anti-mouse IFN-γ mAbs (BD Biosciences, San Diego, CA, USA). Dead cells were stained with AmCyan-conjugated fixable viability dye (eBioscience, San Diego, CA, USA) and excluded from the analysis. Flow cytometry data were acquired on a FACSCanto II and analyzed with FACS Diva software (BD Biosciences, San Diego, CA, USA).

### DC Cytokine Secretion

To analyze the cytokine secretion profile of the intestinal dendritic cells (DCs), CD11c^+^ cells were isolated by magnetic separation from intestinal single cell suspensions and stimulated *in vitro* with 1 µg/mL LPS for 20 h. IL-1β, IL-6, TNF-α, and IL-23 in the cell culture supernatants were quantitated with a BD cytometric bead array (CBA from BD Biosciences, CA, USA). The data were analyzed with FCAP-Array software v1.0.1 (Soft Flow, St. Louis Park, MN, USA).

### DC-iNKT Cell Coculture Experiments

To assess the capacity of intestinal DCs to induce iNKT cell expansion and iNKT17 cell differentiation *in vitro*, CD11c^+^ cells were purified from single cell suspension obtained from intestinal tissues by magnetic separation. iNKT cells were isolated from total splenocytes by staining with PE anti-mouse PBS57/Dimerix and magnetic separation with anti-PE MicroBeads (Miltenyi Biotec, Bologna, Italy). Intestinal DCs were cocultured with purified iNKT cells for 7 days at 1:1 ratio with the addition of IL-7 and IL-15 (10 ng/mL) every 48 h. After 7 days, the cytokine secretion profile of iNKT cells was determined by FACS analysis.

### Microbiota Analysis

Total bacterial DNA was isolated from the mucosa and luminal content of the large intestine of NOD mice and BALB/c mice using PowerFecal™ DNA Isolation kit (Qiagen, Hilden, Germany) following the manufacturers’ instructions. The V3- V4- V5- region of the 16S rRNA gene was amplified using universal primers. The analysis of microbiota metabolically active was performed by pyrosequencing of rRNA cDNA 16S (GS Junior, Roche, Roche Diagnostics GmbH, Mannheim, Germany). Sequences with a high-quality score were used for the taxonomic analysis with QIIME (Quantitative Insights Into Microbial Ecology version 1.6).

### Statistical Analysis

Statistical significances of the differences in the percentages of positive cells were calculated by unpaired two-tailed Student’s *t*-test using GraphPad Prism software. Statistical analysis of the microbiota profiling data was performed on the proportional representation of the taxa (summarized to phyla, class, order, family, and genus levels) using one-way ANOVA test with Bonferroni’s correction. For all tests, a *p* value <0.05 was considered statistically significant.

## Results and Discussion

**Figure 1:**
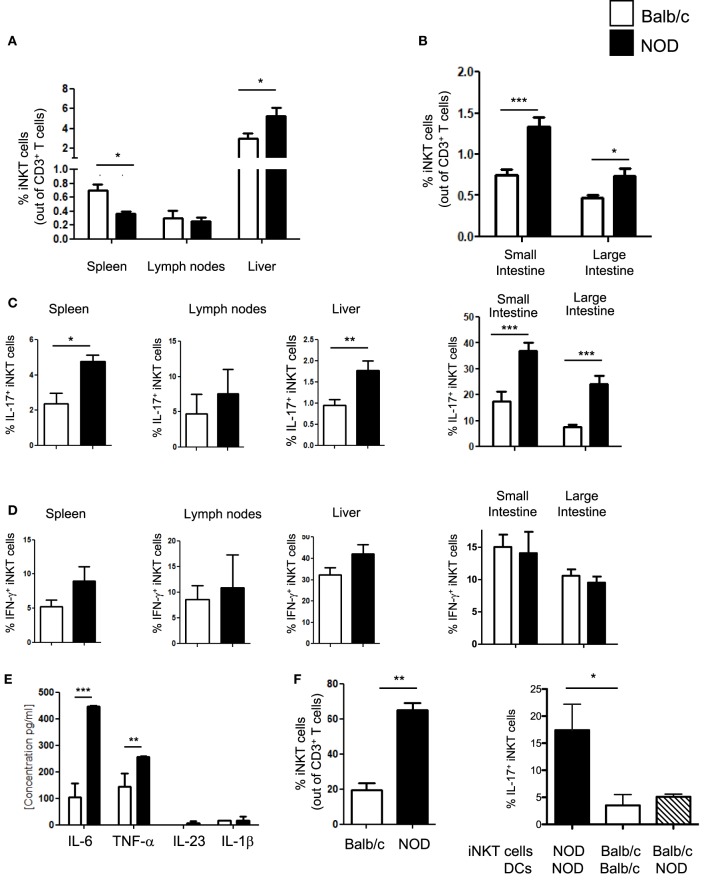
Increased frequency of iNKT17 cells in the spleen, liver, and intestine of non-obese diabetic (NOD) mice. **(A)** Measurements of percentages of total iNKT cells in spleens, lymph nodes, and liver of female 6-week-old Balb/c and NOD mice (8 mice/group). Single cell suspensions obtained from different organs of age- and sex-matched NOD and Balb/c female mice were stained with αGalCer-loaded CD1d tetramers (PBS57-DimersX) in combination with anti-TCR-β monoclonal antibodies and FACS analyzed. Data are presented as mean percentage ± SEM of iNKT cells (PBS57-DimerX^+^TCRβ^+^) out of total TCRβ^+^ T cells. **(B)** Small and large intestines were digested with collagenase D and DNase I and single cell suspensions were obtained by Percoll gradient. Cells were stained and analyzed as in **(A)**. **(C,D)** Total single cell suspensions obtained from the different tissues were stimulated with phorbol 12-myristate 13-acetate/ionomycin for 2.5 h, stained with PBS57-CD1d tetramers in combination with anti-TCR-β monoclonal antibody, then fixed and permeabilized and stained with anti-IL-17 **(C)** or anti-IFN-γ **(D)** monoclonal antibody. Data are expressed as mean percentage ± SEM of IL-17^+^PBS57-DimerX^+^TCRβ^+^ or IFN-γ^+^PBS57-DimerX^+^TCRβ^+^ out of total iNKT cells (PBS57-DimerX^+^TCRβ^+^). **(E)** Cytokine secretion profiles of intestinal dendritic cells (DCs) from NOD and Balb/c mice. Intestinal DCs were purified from the large intestine of NOD or Balb/c mice by magnetic bead separation with anti-CD11c mAb and stimulated *in vitro* with LPS (1 µg/mL). After 20 h, supernatants were collected and cytokine secretion was quantified by cytometric bead array. **(E,F)** Intestinal DCs from NOD mice were more efficient in inducing iNKT cell expansion and iNKT17 cell differentiation. iNKT cells were isolated from splenocytes of BALB/c and NOD mice by magnetic separation and stimulated *in vitro* with αGalCer-pulsed DCs obtained from the intestinal tissues of NOD or Balb/c mice as specified. After 7 days, the cells were collected and FACS analyzed for expression of iNKT cell markers (PBS57-DimersX and anti-TCR-β monoclonal antibodies) and intracellular stained for IL-17. Data are presented as mean percentage ± SEM of iNKT cells out of total TCRβ^+^ T cells (left panel) or IL-17^+^PBS57-DimerX^+^TCRβ^+^ out of total iNKT cells (right panel). The *p* values were calculated using a paired Student’s *t* test. *p < 0.05, **p < 0.01, ***p < 0.001.

iNKT cell with an IL-17-secreting phenotype (iNKT17 cells) have a crucial pathogenic role in autoimmune T1D (6). In female, NOD mice that spontaneously develop T1D, although the overall iNKT cell number is reduced, there is a selective increase in the iNKT17 cell percentage possibly due to their enhanced thymic differentiation (8). Since recent evidence indicates that the intestine is important for iNKT cell maturation and functional differentiation (10), we used female NOD mice with an enlarged iNKT17 cell repertoire to ask whether the gut mucosa is a preferential site for iNKT17 cell expansion. Total iNKT cells and iNKT17 cells were analyzed in the peripheral lymphoid organs (spleen and peripheral lymph nodes), liver and small and large intestine of NOD and control non-autoimmune Balb/c mice. Secretion of IL-17 and IFN-γ by iNKT cells (iNKT17 and iNKT1 cell phenotypes) was detected after staining with αGalCer-loaded CD1d tetramers and stimulation with PMA and ionomycin. Our analysis confirmed a significantly reduced percentage (Figure 1A) and absolute number (Figure S1 in Supplementary Material) of iNKT cells in the spleen of NOD mice. On the contrary, the iNKT cell presence in the intestinal tissues as well as in the liver was augmented in NOD mice compared to control Balb/c mice both in terms of relative percentages (Figures 1A,B) and absolute numbers (Figure S1 in Supplementary Material). We also confirmed that, in NOD mice, there is a selective expansion of the iNKT17 cell subset in all organs analyzed (Figure 1C). However, we noted that the increase in iNKT17 cell frequency in NOD mice was much more significant in the intestinal tissues (particularly in the large intestine) in comparison with peripheral lymphoid organs and liver. In fact, while there is a twofold increase of iNKT17 cells in the spleen, liver, and small intestine of NOD mice in comparison with control Balb/c mice, we detected a fourfold increase in the large intestine of NOD mice (Figure 1C). The frequency of other iNKT cell subsets (iNKT1 cells) was similar between NOD and control Balb/c mice in all organs analyzed (Figure 1D). These results suggest that there is a preferential expansion of iNKT17 cells in the large intestine of NOD mice.

Next, we asked whether increased intestinal iNKT17 cell frequency in NOD mice is due to the presence in their gut mucosa of pro-inflammatory dendritic cells (DCs) that preferentially drive iNKT cells toward an IL-17-secreting iNKT17 phenotype. The factors that regulate peripheral iNKT17 cell differentiation have not yet been characterized, but we hypothesized that DC release of Th17-priming cytokines such as IL-6 and IL-23 could also drive iNKT17 cell differentiation (17, 18). In line with this notion, we found that intestinal DCs of NOD mice secrete higher amount of IL-6 in comparison with their counterparts from control Balb/c mice (Figure 1E). Moreover, intestinal DCs of NOD mice were more efficient in promoting iNKT cell expansion (Figure 1F, left panel) and inducing iNKT17 cell differentiation *in vitro* compared to intestinal DCs of Balb/c mice (Figure 1F, right panel). However, intestinal DC are not directly responsible for iNKT17 cell differentiation since stimulation of iNKT cells from Balb/c mice with intestinal NOD DCs was not sufficient to drive them toward an iNKT17 cell phenotype (Figure 1F, right panel). We concluded that intestinal iNKT cells of NOD mice were intrinsically more prone to acquire a biased iNKT17 cell phenotype than iNKT cells from Balb/c mice.

**Figure 2:**
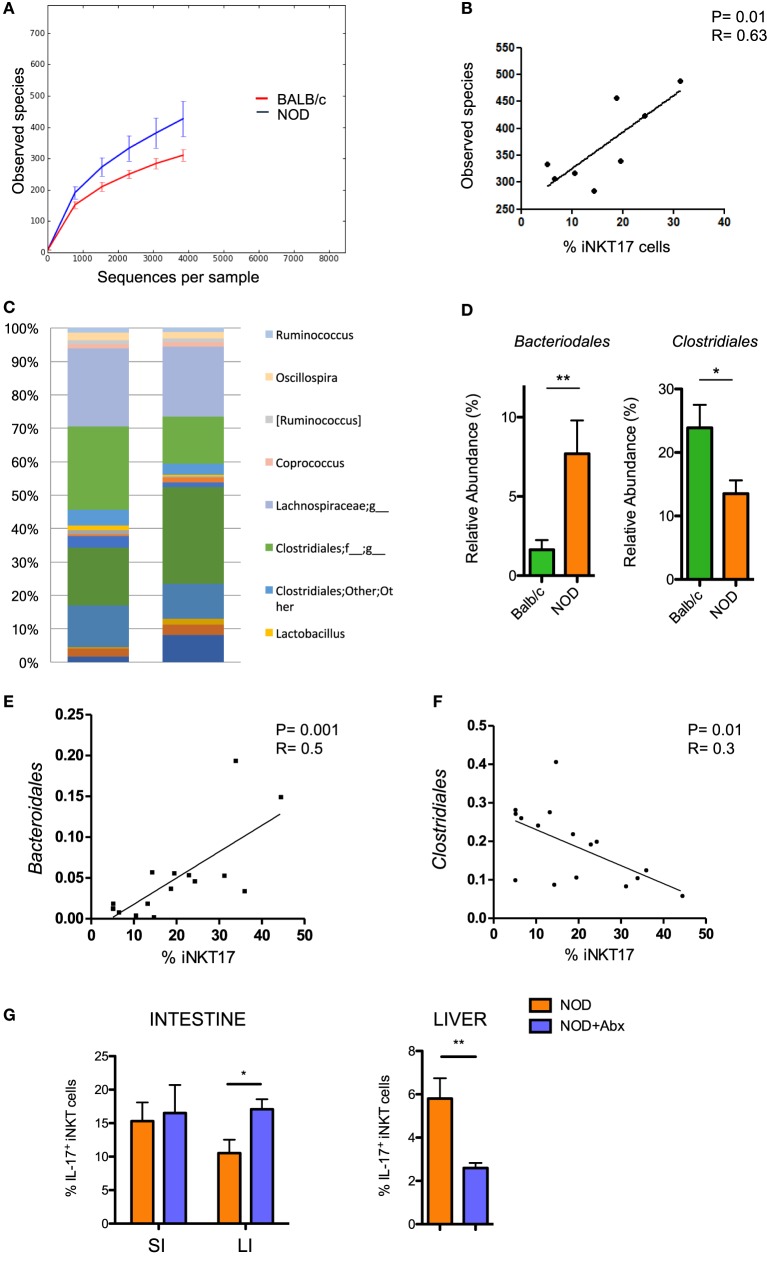
Increased intestinal iNKT17 cell frequency in non-obese diabetic (NOD) mice correlates with the commensal microbiota composition. Microbiota profiling was performed on microbiota samples obtained from the lumen and mucosal tissue of the large intestine of female 6-week-old Balb/c and NOD mice (8 mice/group). 16S mRNA analysis and QIIME software were used for the analysis of microbiota composition and species distribution between the two groups. **(A)** Alpha diversity of the commensal microbiota composition isolated of NOD mice and BALB/c mice. **(B)** Correlation between total number of bacterial species detected in the large intestine and percentage of intestinal iNKT17 cells in NOD mice. **(C)** Profiles of the commensal microbiota composition at the genus level in Balb/c and NOD mice. **(D)** Relative abundances of *Bacteriodales* and *Clostridiales* strains in Balb/c and NOD mice. Data are presented as mean percentage ± SEM of the relative abundance of the different bacterial strains in the two murine strains. **(E,F)** Correlative analyses between the relative abundance of *Bacteroidales* or *Clostridiales* strains and the frequency of iNKT17 cells in the large intestine of NOD mice. Statistical analysis of the microbiota profiling data was performed by using one-way ANOVA test with Bonferroni’s correction. **(G)** Percentages of iNKT17 cells in intestinal the intestine and liver changes upon antibiotic treatment. 5-week-old NOD mice were treated with broad-spectrum antibiotics (ampicillin, 1 g/L; neomycin 1 g/L; metronidazole, 1 g/L; vancomycin, 0.5 g/L) for 1 week in drinking water. Single cell suspensions were obtained from different tissues, stimulated with phorbol 12-myristate 13-acetate + ionomycin for 4 h, stained with PBS57-CD1d tetramers in combination with anti-TCR-β monoclonal antibody, then fixed and permeabilized and stained with anti-IL-17 monoclonal antibody. Data are expressed as mean percentage ± SEM of IL-17^+^PBS57-DimerX^+^TCRβ^+^ out of total iNKT cells (PBS57-DimerX^+^TCRβ^+^). *p < 0.05, **p < 0.01.

Having established that the frequency of iNKT17 cells is increased in the intestine of NOD mice, we wanted to highlight the mechanism responsible for this effect. The composition of the gut commensal microbiota can have a strong impact on iNKT17 cell expansion. To explore this possibility, we analyzed the profiles of the intestinal microbial community by ultra-deep pyrosequencing of barcoded 16S rRNA gene amplicons on samples obtained from the luminal content and mucosa of the large intestines of NOD mice and Balb/c mice. Our taxonomic, functional, and diversity microbiome profiling revealed significant differences in the gut commensal microbiota composition in NOD mice vs control Balb/c mice. Specifically, we found that NOD mice have a higher bacterial richness (Figure 2A) that directly correlates with iNKT17 cell percentages in the large intestine (Figure 2B). At the genus level, we demonstrated that the microbiota composition in the large intestine of NOD mice is very different compared to non-autoimmune Balb/c mice (Figure 2C) with a selective increase in the relative abundance of *Bacteroidales* and reduction of *Clostridiales* strains (Figure 2D). Importantly, we found that in the NOD mice the iNKT17 cell frequency positively correlates with the relative abundance of *Bacteroidales* species (*p* > 0.01) (Figure 2E) and inversely correlates with the presence of *Clostridiales* strains (*p* *< 0.05*) (Figure 2F). To furtherly highlight the role of the gut microbiota in iNKT17 cell expansion, we treated NOD mice with broad-spectrum antibiotics and measured the percentages of iNKT17 cells in the gut mucosa and systemically (liver tissue). Our data show statistically significant alterations in the percentages of iNKT17 cells in the liver and in the intestine of antibiotic-treated NOD mice compared to untreated NOD controls (Figure 2G). However, we observed that iNKT17 cell frequency decreased in the liver but increased in the intestinal tissue. This discrepancy may be related to the uncomplete deletion of endogenous commensal microbiota induced by the antibiotic treatment in NOD mice (data not shown). This could have favored microbial species that negatively regulate iNKT17 cells in the liver (probably by passing through the intestinal barrier into the systemic circulation) and other species that positively affect iNKT17 cell expansion in the gut mucosa.

Our data suggest that the intestinal environment of NOD mice regulates iNKT17 cell differentiation though gut microbiota modification. Previous reports have shown that microbial regulation of iNKT cell expansion and functional maturation extends beyond the intestinal compartments. For example, in germ-free mice, iNKT cells are hyporesponsive not only in the gut mucosa but also in peripheral lymphoid organs (10). Immune cells, including iNKT cells, after being modulated in the gut, travel to secondary lymphoid organs and peripheral tissues (19). Hence, the gut microbiota composition of NOD mice could promote T1D by favoring intestinal expansion of effector iNKT17 cells that from the gut mucosa move to pancreatic lymph nodes and tissues to promote T1D pathogenesis.

## Ethics Statement

All mice were maintained under specific pathogen-free conditions in the animal facility at San Raffaele Scientific Institute and all experiments were conducted in accordance with the Institutional Animal Care and Use Committee.

## Author Contributions

LDG, CS, and IC performed all *in vivo* and *in vitro* experiments. LDG analyzed data and prepared manuscript’s figures. RF and FC performed microbiome analysis. MF served as principal investigator, analyzed data, and wrote the manuscript.

## Conflict of Interest Statement

The authors declare that the research was conducted in the absence of any commercial or financial relationships that could be construed as a potential conflict of interest.
