# Supplementary material for: Increased iNKT17 Cell Frequency in the Intestine of Non-Obese Diabetic Mice Correlates With High Bacterioidales and Low Clostridiales Abundance
Source: Front Immunol. 2018 Jul 30;9:1752. doi: 10.3389/fimmu.2018.01752 (PMC6077215; doi:10.3389/fimmu.2018.01752)
Supplement: Supplementary file 1 [file data_sheet_1.PDF]

Figure S1

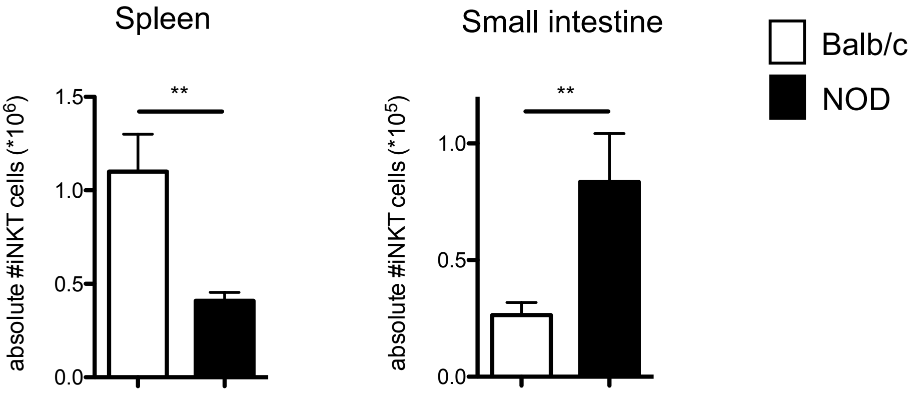

Supplementary Figure 1. Absolute numbers of iNKT cells in the spleen and intestinal mucosa of female 6 week-old NOD mice and sex- and age-matched Balb/c controls. Single cell suspensions obtained from spleen or intestinal mucosa were counted and stained with αGalCer-loaded CD1d tetramers (PBS57-DimersX) in combination with anti-TCR-β monoclonal antibodies and FACS analysed. Total numbers of iNKT cells were calculated based on the percentages of PBS57-DimerX$^+$TCRβ$^+$ out of total cells. Data are expressed as mean ± SEM. The *p* values were calculated using a paired Student *t* test. *$p < 0.05$, **$p < 0.01$, ***$p < 0.001$.

Figure S2

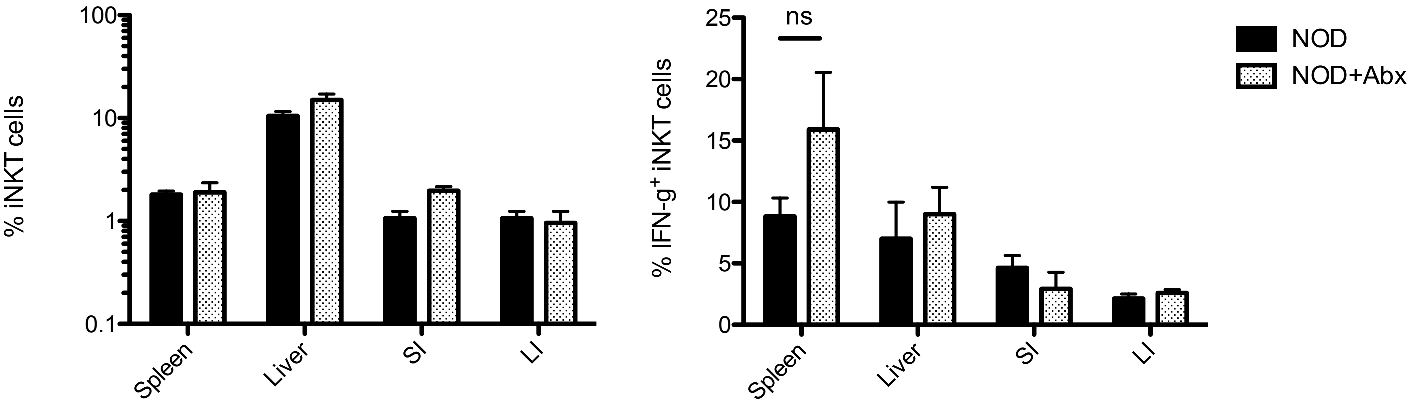

Supplementary Figure 2. Percentages of iNKT cells and iNKT1 cells in the peripheral organs and intestinal mucosa of NOD mice treated with broad-spectrum antibiotics. 5 week-old NOD mice were treated with antibiotics (ampicillin, 1 g/L; neomycin 1 g/L; metronidazole, 1 g/L; vancomycin, 0.5g/L) for one week in drinking water. Single cell suspension were obtained from different tissues were stimulated with PMA/Ionomycin for 4 hours, stained with PBS57-CD1d tetramers in combination with anti-TCR-β monoclonal antibody then fixed and permeabilized and stained with anti-IFN-γ monoclonal antibody. Data are expressed as mean percentage ± SEM of iNKT cells (PBS57-DimerX$^+$TCRβ$^+$) out of total T cells (TCRβ$^+$ cells) or IFN-γ$^+$PBS57-DimerX$^+$TCRβ$^+$ out of total iNKT cells.
